# Impact of ACEIs and ARBs-related adverse drug reaction on patients’ clinical outcomes: a cohort study in UK primary care

**DOI:** 10.3399/BJGP.2023.0153

**Published:** 2023-10-03

**Authors:** Widya N Insani, Cate Whittlesea, Chengsheng Ju, Kenneth KC Man, Matthew Adesuyan, Sarah Chapman, Li Wei

**Affiliations:** Research Department of Practice and Policy, School of Pharmacy, University College London, London, UK; Centre of Excellence for Pharmaceutical Care Innovation, Department of Pharmacology and Clinical Pharmacy, Padjadjaran University, Bandung, Indonesia.; Research Department of Practice and Policy, School of Pharmacy, University College London, London, UK.; Research Department of Practice and Policy, School of Pharmacy, University College London, London, UK.; Research Department of Practice and Policy, School of Pharmacy, University College London, London, UK; Laboratory of Data Discovery for Health, Hong Kong Science Park, Hong Kong Speical Administrative Region, China.; Research Department of Practice and Policy, School of Pharmacy, University College London; Centre for Medicines Optimisation Research and Education, University College London Hospitals NHS Foundation Trust, London, UK.; Institute of Pharmaceutical Science, King’s College London, London, UK.; Research Department of Practice and Policy, School of Pharmacy, University College London; Centre for Medicines Optimisation Research and Education, University College London Hospitals NHS Foundation Trust, London, UK; Laboratory of Data Discovery for Health, Hong Kong Science Park, Hong Kong Speical Administrative Region, China.

**Keywords:** adverse drug reaction, drug-related side effects and adverse reactions, primary health care

## Abstract

**Background:**

Adverse drug reaction (ADR) related to angiotensin-converting enzyme inhibitors (ACEIs) and angiotensin receptor blockers (ARBs) may negatively affect patients’ treatment outcomes.

**Aim:**

To investigate the impact of ACEIs/ARBs-related ADR consultation on cardiovascular disease (CVD) events and all-cause mortality.

**Design and setting:**

Propensity score-matched cohort study of ACEIs/ARBs between 2004 and 2019 using UK IQVIA medical research data.

**Method:**

ADR consultations were identified using standardised designated codes. Propensity scores were calculated based on comorbidities, concomitant medications, frailty, and polypharmacy. Cox’s proportional hazard regression model was used to compare the outcomes between patients in ADR and non-ADR groups. In the secondary analysis, treatment- pattern changes following the ADR were examined and the subsequent outcomes were compared.

**Results:**

Among 1 471 906 eligible users of ACEIs/ARBs, 13 652 (0.93%) patients had ACEIs/ARBs- related ADR consultation in primary care. Patients with ACEIs/ARBs-related ADR consultation had an increased risk of subsequent CVD events and all- cause mortality in both primary prevention (CVD events: adjusted hazard ratio [aHR] 1.22, 95% confidence interval [CI] = 1.05 to 1.43; all-cause mortality: aHR 1.14, 95% CI = 1.01 to 1.27) and secondary prevention cohorts (CVD events: aHR 1.13, 95% CI = 1.05 to 1.21; all-cause mortality: aHR 1.15, 95% CI = 1.09 to 1.21). Half (50.19%) of patients with ADR continued to use ACEIs/ARBs, and these patients had a reduced risk of mortality (aHR 0.88, 95% CI = 0.82 to 0.95) compared with those who discontinued using ACEIs/ARBs.

**Conclusion:**

This study provides information on the burden of ADR on patients and the health system. The findings call for additional monitoring and treatment strategies for patients affected by ADR to mitigate the risks of adverse clinical outcomes.

## INTRODUCTION

Angiotensin-converting enzyme inhibitors (ACEIs) and angiotensin receptor blockers (ARBs) are two renin- angiotensin- aldosterone system inhibitors (RAASIs) that are among the most frequently prescribed drugs worldwide.^1^^–^3 These medications are commonly indicated for a number of conditions, including hypertension, chronic kidney diseases (CKDs), and heart failure (HF).^4^^–^9 Treatment with ACEIs/ARBs has been shown to reduce morbidity and mortality.^10^^–^12

Previous studies have reported that up to 3.9% of users of ACEIs/ARBs may develop adverse drug reactions (ADRs), including persistent dry cough, hyperkalaemia, dizziness, hypotension, gastrointestinal symptoms, palpitation, excessive urination, and angioedema.^13^^,^^14^ A UK-based study by Tsang *et al* found that ACEIs were among the most common drug class involved in ADRs in primary care.^15^ Risk of ACEIs/ARBs-related ADR increased with dual RAASI combinations, history of smoking, progression of CKD stages, hypoaldosteronism, and the use of concomitant medication, such as other antihypertensive drugs, non-steroidal anti- inflammatory drugs (NSAIDs), heparin, and immunosuppressants.^16^^–^19

In addition to direct physiological impact, ADRs may have negative consequences on patients’ treatment outcomes.^20^ Previous studies have shown that up to one-third of patients with hypertension had their treatment reduced and/or interrupted owing to ADRs, which precluded treatment options to achieve their blood pressure target.^14^^,^^21^ Clinical guidelines indicate that, depending on the severity of the reactions and underlying comorbidities, management of ACEIs/ARBs- related ADR may vary between patients, including: altering the dosage regimen; switching between ACEIs/ARBs and/or other drug classes; and ascertaining the necessary monitoring, for example, that of renal function and electrolytes.^22^^,^^23^

**Table:** How this fits in

Adverse drug reactions (ADRs) represent a considerable burden for patients and the healthcare system. ADRs related to angiotensin-converting enzyme inhibitors (ACEIs) and angiotensin receptor blockers (ARBs) were among the most frequent ADRs documented in primary care records; however, there is limited information on the impact of ACEI/ARB-related ADRs on patient outcomes and changes to treatment patterns post-ADR in this setting. This study found that ACEI/ARB-related ADR consultations were associated with subsequent major cardiovascular events and all-cause mortality, indicating that the affected patients should be monitored more closely by healthcare professionals to mitigate the risk of adverse clinical outcomes.

There is limited information on the impact of ACEIs/ARBs-related ADR on patients’ clinical outcomes. Such findings may help to improve understanding of the burden of ADRs, and better inform patient care and monitoring for individuals at high risk of untoward clinical outcomes. The objectives of this study were to examine the impact of ACEIs/ARBs-related ADR consultation on subsequent cardiovascular disease (CVD) events and all-cause mortality, and investigate treatment-pattern changes following these ADRs in UK primary care settings.

## METHOD

### Data source

This study was conducted using IQVIA Medical Research Data UK that incorporates data from The Health Improvement Network (THIN).^24^ The data contains de-identified information provided by patients as part of their routine primary care. UK primary care databases have been used previously to investigate ADR-related consultations.^15^^,^^25^^–^27

### Study design

This cohort study included patients who used ACEIs/ARBs between 2004 and 2019. Patients were excluded if they:

- had a missing date of birth or sex;
- were aged <18 years at the date of first ACEI/ARB prescription;
- had an ACEI/ARB-related ADR before 2004;
- were registered <1 year before the index date; or
- had a history of cancer.

As a history of CVD increases the risk of recurrent CVD events and mortality, the analysis was stratified based on CVD primary prevention and secondary prevention — that is, without and with a history of CVD, respectively. CVD was defined as coronary heart disease (angina and myocardial infarction), cerebrovascular disease (stroke and transient ischaemic attack [TIA]), and peripheral arterial disease. Patients with HF were included in the secondary prevention because of their level of risk being equivalent to that of people with an established CVD.^28^^,^^29^ The study design is presented in Supplementary Figure S1.

### Exposed cohort

The exposed cohort comprised patients with an ACEI/ARB-related ADR consultation in primary care. ‘ACEI/ARB- related ADR consultation’ was defined using standardised designated codes, for example, Read code chapter TJ (adverse drug reactions), as previously examined.^15^^,^^25^^–^27 As this study used designated codes specific to ACEIs/ARBs- related ADR consultation, it was estimated that the ADR consultation was attributed to ACEI/ARB therapy. The index date was defined as the date of the first ACEI/ARB-related ADR consultation (see Supplementary Box S1).

### Control cohort

The control cohort comprised users of ACEIs/ARBs, who did not have an ACEI/ARB- related ADR consultation in primary care. To generate a control cohort, an index date was assigned at random to a sample of 30% of unexposed patients — that is, those without an ACEI/ARB-related ADR — by incidence density sampling from the distribution of index dates in the exposed cohort.^30^ After excluding patients who died or transferred before, or at, the index date, or had been registered for <1 year, or had history of cancer, propensity score matching (1:1) was used to select the control group using the greedy matching algorithm.^31^ Patients with a history of any cancer were excluded, as cancers negatively affect survival.

### Covariates

The covariates measured were:

- age;
- sex;
- interval between the ACEI/ARB initiation date and the index date;
- comorbidities: hypertension, CKD, type 1 and type 2 diabetes mellitus, dyslipidaemia, chronic liver disease, chronic obstructive pulmonary disease, and rheumatic disease — recorded at any time before, or on, the index date;
- use of concomitant medications: calcium channel blockers (CCBs), diuretics, beta-blockers, statins, antiplatelets/anticoagulants, antidiabetics, nitrates, and NSAIDs — recorded ≤180 days before the index date;
- electronic frailty index (eFi), comprising 36 health conditions as developed and validated by Clegg *et al*;^32^ and
- polypharmacy.

Frailty index categories were: fit, mild, moderate, and severe frailty. Polypharmacy was defined as the use of between five and nine medications; excessive polypharmacy was defined as the use of ≥10 medications.^33^

### Propensity score

The propensity score was defined as the probability of receiving the exposure (ACEI/ARB-related ADR), which was estimated using a logistic regression model based on all covariates at baseline.^34^ It was used to control for confounding due to non-randomised exposure allocation, by generating a comparable distribution of measured covariates across exposed and control groups. In the matched sample, the balance of covariates was assessed using standardised mean difference (SMD). An SMD of <0.2 indicated a negligible difference in covariates between both groups.^35^

### Outcomes and follow-up period

The primary outcome was the first composite CVD events (myocardial infarction and stroke/TIA), and the secondary outcome was all-cause mortality. The follow-up for each patient commenced from the date of ACEI/ARB-related ADR or the index date until the occurrence of the outcome or any censoring event (patient transferred out, death, study end date), whichever was earlier.

### Secondary, subgroup, and sensitivity analysis

In the secondary analysis, treatment-pattern changes within 12 months following the ADR consultation were examined and the subsequent outcomes were compared. The continued ACEI/ARB prescription was defined as any prescription within 12 months after the ADR consultation, as used in a previous study examining continued drug prescription following ADRs.^36^ Patients who died, transferred, had their last day of follow-up, or CVD events within 1 year after the ADR date were excluded to reduce immortal time bias. The eligible patients were classified as:

- continued ACEIs/ARBs — either continuing the current treatment or switching to another ACEI/ARB; or
- discontinued ACEIs/ARBs.

The subsequent outcomes were compared between those who continued and discontinued using ACEIs/ARBs following the ADR using stabilised inverse probability of treatment weighting (IPTW), with the propensity score estimated from all covariates (as in the main analysis). The follow-up commenced from 12 months following the ADR until the earliest of the following: outcome of interest, patient transferred out, death, or study end date. A competing risk analysis was performed using Fine–Gray’s subdistribution hazard model.^37^

Subgroup analyses were performed separately based on different indications for ACEIs/ARBs — that is, hypertension, CKD, and HF. A sensitivity analysis using stabilised IPTW was conducted for the primary analysis. The window period to examine treatment changes in the secondary analysis was adjusted from 12 to 6 months to evaluate robustness.^26^

As UK clinical guidelines consider ethnicity differences for the selection of antihypertensive drugs, including ACEIs/ARBs, a separate sensitivity analysis was conducted for those patients for whom ethnicity data were available. An additional analysis among those who continued using ACEIs/ARBs in the ADR group versus the control group was conducted to examine whether the continuation of ACEIs/ARBs affected the outcomes.

### Statistical analyses

Baseline characteristics were expressed as frequencies and percentages for categorical variables, and as means with standard deviations (SDs) for continuous variables. Cox’s proportional hazard regression model and the Kaplan–Meier method were used to estimate the risk of CVD events and all-cause mortality. The results were presented as adjusted hazard ratios (aHRs) with 95% confidence intervals (CIs). A two- sided *P*-value <0.05 was considered statistically significant. Analyses were performed using SAS (version 9.4).

## RESULTS

### Baseline characteristics

During the study period (2004–2019), 1 513 241 users of ACEIs/ARBs were identified; after exclusion, 1 471 906 patients were eligible to be included in the analysis. Of these, 13 652 (0.93%) patients had an ACEI/ARB-related ADR consultation in primary care. The flowchart of the participant selection process is shown in Supplementary Figure S2.

The mean ages were 68.11 years (SD 13.28) and 74.58 years (SD 10.91) for the CVD primary (*n* = 6196) and secondary (*n* = 14 238) prevention cohorts, respectively (Table 1). After matching, the SMD of all covariates was <0.2, indicating comparability between the ADR consultation and non-ADR consultation groups in both the primary and secondary prevention cohorts. The baseline characteristics before and after propensity score matching are given in Supplementary Table S1.

**Table 1.** Baseline characteristics of the participants

**Characteristics**	**CVD primary prevention matched cohort, *n* (%)^a^**			**CVD secondary prevention matched cohort, *n* (%)^a^**		

	**With ACEI/ARB-related ADR consultation**	**Without ACEI/ARB-related ADR consultation**	**SMD^b^**	**With ACEI/ARB-related ADR consultation**	**Without ACEI/ARB-related ADR consultation**	**SMD^b^**
**Total**	3098	3098		7119	7119	

**Mean age, years (±SD)**	68.11 (13.38)	68.11 (13.18)	−0.0006	74.58 (10.96)	74.59 (10.86)	−0.0008

**Sex, male**	1210 (39.06)	1177 (37.99)	0.0219	3712 (52.14)	3741 (52.55)	−0.0082

**Mean interval between commencement of ACEI/ARB therapy and index date, years (±SD)**	3.60 (3.80)	3.60 (2.98)	−0.0003	5.04 (4.41)	4.98 (3.74)	0.0161

**Comorbidities**						
Hypertension	2728 (88.06)	2739 (88.41)	−0.0110	5038 (70.77)	5019 (70.50)	0.0059
Dyslipidaemia	528 (17.04)	508 (16.40)	0.0173	1809 (25.41)	1796 (25.23)	0.0042
Diabetes	1367 (44.13)	1393 (44.96)	−0.0169	2462 (34.58)	2444 (34.33)	0.0053
CKD	1333 (43.03)	1301 (41.99)	0.0209	2826 (39.70)	2808 (39.44)	0.0052
Liver disease	44 (1.42)	49 (1.58)	−0.0133	74 (1.04)	77 (1.08)	−0.0041
COPD	287 (9.26)	287 (9.26)	0.0000	1280 (17.98)	1266 (17.78)	0.0051
Rheumatic disease	360 (11.62)	348 (11.23)	0.0122	1263 (17.74)	1272 (17.87)	−0.0033

**Concomitant medications**						
CCBs	1268 (40.93)	1254 (40.48)	0.0092	2432 (34.16)	2453 (34.46)	−0.0062
Diuretics	1534 (49.52)	1517 (48.97)	0.0110	4221 (59.29)	4166 (58.52)	0.0157
Beta-blockers	790 (25.50)	814 (26.28)	−0.0177	3533 (49.63)	3588 (50.40)	−0.0155
Statins	1342 (43.32)	1298 (41.90)	0.0287	5145 (72.27)	5199 (73.03)	−0.0170
Antiplatelets/anticoagulants	974 (31.44)	971 (31.34)	0.0021	5425 (76.20)	5464 (76.75)	−0.0129
Antidiabetics	1002 (32.34)	1036 (33.44)	−0.0234	1691 (23.75)	1685 (23.67)	0.0020
Nitrates	47 (1.52)	39 (1.26)	0.0221	2402 (33.74)	2420 (33.99)	−0.0053
NSAIDs	427 (13.78)	432 (13.94)	−0.0047	747 (10.49)	730 (10.25)	0.0078

**Electronic Frailty Index**			0.0509			0.1089
Fit	2090 (67.46)	2075 (66.98)		2438 (34.25)	2304 (32.36)	
Mild	843 (27.21)	882 (28.47)		2928 (41.13)	3263 (45.84)	
Moderate	146 (4.71)	133 (4.29)		1403 (19.71)	1245 (17.49)	
Severe	19 (0.61)	8 (0.26)		350 (4.92)	307 (4.31)	

**Polypharmacy**			0.0241			0.0360
No polypharmacy	814 (26.28)	816 (26.34)		715 (10.04)	664 (9.33)	
Polypharmacy (5–9 medications)	1453 (46.90)	1421 (45.87)		2908 (40.85)	2996 (42.08)	
Excessive polypharmacy (≥10 medications)	831 (26.82)	861 (27.79)		3496 (49.11)	3459 (48.59)	

a *Unless otherwise stated.*

b *SMD indicates difference in mean or proportion of covariates in the exposed versus control group, divided by the pooled standard deviation. SMD <0.2 indicates a negligible difference in covariates between both groups. ACEI = angiotensin-converting enzyme inhibitor. ADR = adverse drug reaction. ARB = angiotensin receptor blocker. CCB = calcium channel blocker. CKD = chronic kidney disease. COPD = chronic obstructive pulmonary disease. CVD = cardiovascular disease. NSAID = non-steroidal anti-inflammatory drug. SD = standard deviation. SMD = standardised mean difference.*

### ACEI/ARB-related ADRs, and the risk of a CVD event and all-cause mortality

#### CVD primary prevention cohort

During the mean follow-up time of 6.57 years (SD 3.96), 648 patients had CVD events: 366/3098 (11.81%) were in the ADR consultation group and 282/3098 (9.10%) were in the control group. Cox regression analysis showed that patients with an ACEI/ARB-related ADR had an increased risk of subsequent CVD event compared with users of ACEIs/ARBs without an ADR (aHR 1.22, 95% CI = 1.05 to 1.43) (Table 2). Similar results were observed for the secondary outcome, all-cause mortality. During the mean follow-up time of 6.93 years (SD 3.96), there were 1196 deaths; of these, 659 (21.27%) were in the ADR consultation group and 537 (17.33%) were in the control group. ACEI/ARB- related ADRs increased the risk of all- cause mortality (aHR 1.14, 95% CI = 1.01 to 1.27) (Table 2).

**Table 2.** Adjusted hazard ratio and incidence rate per 1000 person–years (95% CI) for CVD events and all- cause mortality in CVD primary and secondary prevention cohorts

**Outcome**	**CVD primary prevention cohort**					**CVD secondary prevention cohort**				
	**ADR group, *N* = 3098**		**Control group, *N* = 3098**			**ADR group, *N* = 7119**		**Control group, *N* = 7119**		
	**Event, *n* (%)**	**Incidence rate (95% CI)**	**Event, *n* (%)**	**Incidence rate (95% CI)**	**aHR (95% CI)**	**Event, *n* (%)**	**Incidence rate (95% CI)**	**Event, *n* (%)**	**Incidence rate (95% CI)**	**aHR (95% CI)**
**Primary outcome**										
Composite CVD events	366 (11.81)	17.46 (15.76 to 19.34)	282 (9.10)	14.27 (12.70 to 16.04)	1.22 (1.05 to 1.43)	1574 (22.11)	46.07 (43.85 to 48.41)	1431 (20.10)	41.12 (39.05 to 43.31)	1.13 (1.05 to 1.21)
Myocardial infarction	140 (4.52)	6.43 (5.44 to 7.58)	98 (3.16)	4.81 (3.95 to 5.86)	1.33 (1.03 to 1.72)	667 (9.37)	17.78 (16.42 to 19.26)	510 (7.16)	12.62 (11.49 to 13.86)	1.32 (1.18 to 1.48)
Stroke/TIA	250 (8.07)	11.71 (10.34 to 13.26)	198 (6.39)	9.85 (8.57 to 11.32)	1.19 (0.98 to 1.43)	1041 (14.62)	28.87 (27.17 to 30.68)	1016 (14.27)	28.13 (26.45 to 29.91)	1.04 (0.95 to 1.13)
**Secondary outcome**										
All-cause mortality	659 (21.27)	29.65 (27.47 to 32.00)	537 (17.33)	25.90 (23.80 to 28.19)	1.14 (1.01 to 1.27)	2792 (39.22)	69.53 (67.00 to 72.16)	2416 (33.94)	60.38 (58.02 to 62.84)	1.15 (1.09 to 1.21)

*ADR = adverse drug reaction. aHR = adjusted hazard ratio. CVD = cardiovascular disease. TIA = transient ischaemic attack.*

#### CVD secondary prevention cohort

During the mean follow-up time of 4.84 years (SD 3.84), 3005 patients had recurrent CVD events in the secondary prevention cohort; 1574/7119 (22.11%) of these occurred in the ADR group and 1431/7119 (20.10%) in the control group. ACEI/ARB-related ADRs were associated with an increased risk of recurrent CVD events (aHR 1.13, 95% CI = 1.05 to 1.21) (Table 2).

Similarly, for the mortality outcome, during the mean follow-up time of 5.63 years (SD 3.95), there were 5208 deaths: 2792 (39.22%) and 2416 (33.94%) in the ADR and control groups, respectively. ACEI/ARB-related ADRs increased the risk of all-cause mortality (aHR 1.15, 95% CI = 1.09 to 1.21) (Table 2 and Supplementary Figure S3).

#### Subgroup analysis among patients with hypertension, CKD, and HF

**Figure 1.**
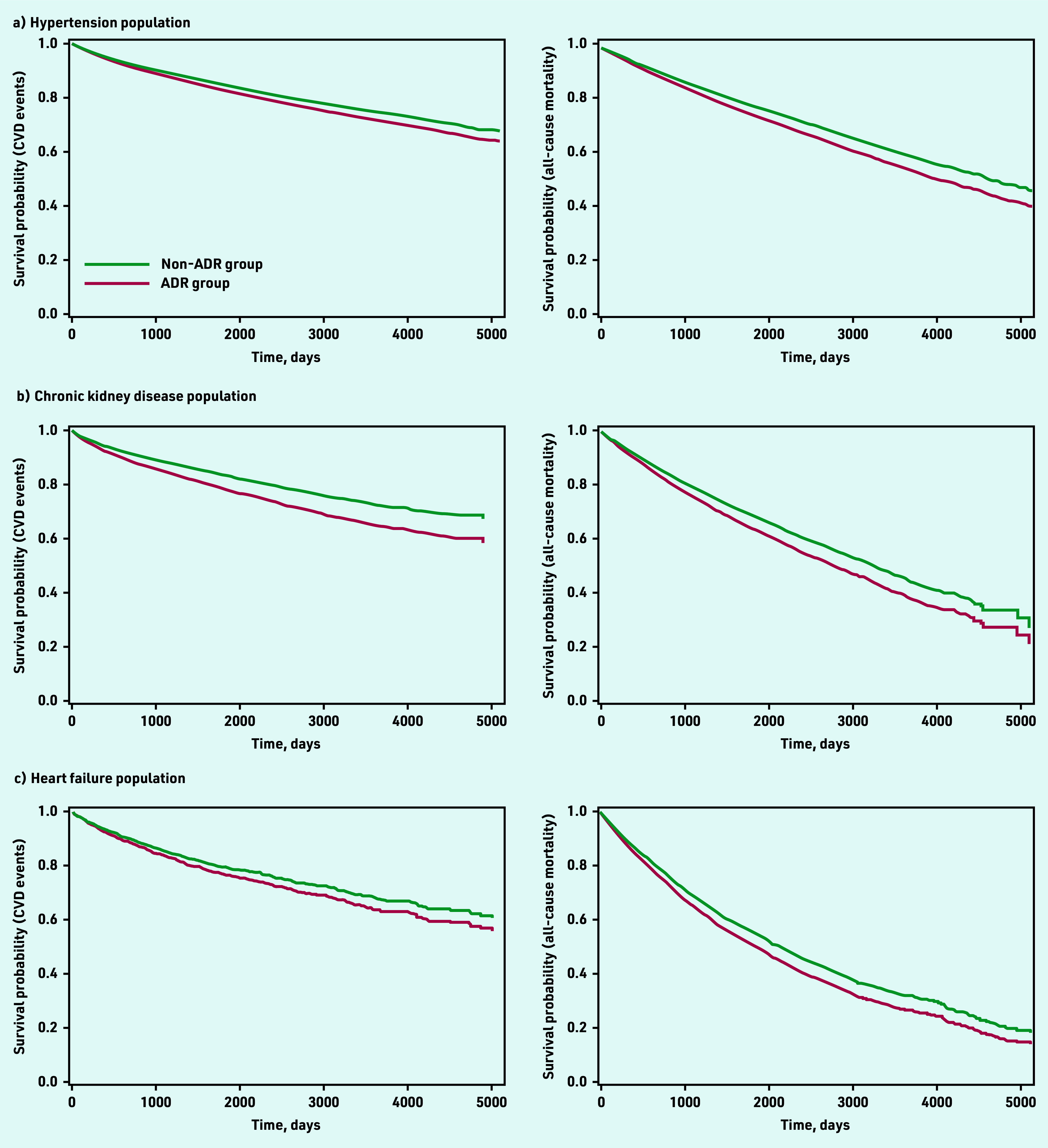
*Kaplan-Meier survival curves comparing survival probability relating to CVD outcomes and all- cause mortality between ADR and non-ADR groups across different indications for ACEIs/ARBs: a) hypertension population; b) chronic kidney disease population; and c) heart failure population. ACEI = angiotensin-converting enzyme inhibitor. ADR = adverse drug reaction. ARB = angiotensin receptor blocker. CVD = cardiovascular disease.*

Patients with hypertension who had ACEI/ARB-related ADRs had an increased risk of subsequent CVD events (aHR 1.13, 95% CI = 1.05 to 1.21) and all-cause mortality (aHR 1.16, 95% CI = 1.09 to 1.22). Consistent findings were observed among patients with CKD; ACEI/ARB-related ADRs were associated with CVD events (aHR 1.35, 95% CI = 1.22 to 1.50) and all-cause mortality (aHR 1.24, 95% CI = 1.16 to 1.33). Patients with HF had the highest incidence rates of CVD events and all-cause mortality when compared with the hypertension and CKD populations; however, patients with HF and ACEI/ARB-related ADRs had a similar risk of CVD events (aHR 1.12, 95% CI = 0.98 to 1.28), but increased risk of all-cause mortality (aHR 1.16, 95% CI = 1.07 to 1.25) compared with those without ADRs (Table 3 and Figure 1).

**Table 3.** Subgroup analysis across different indications for angiotensin-converting enzyme inhibitors or angiotensin receptor blockers

**Outcomes**	**ADR group**		**Control group**		**aHR (95% CI)**
	**Event, *n* (%)**	**Incidence rate (95% CI)**	**Event, *n* (%)**	**Incidence rate (95% CI)**	
**Patients with hypertension (*n* = 141 151)^a^**					
Composite CVD events	1494 (19.28)	35.44 (33.69 to 37.29)	1334 (17.21)	31.67 (30.01 to 33.41)	1.13 (1.05 to 1.21)
All-cause mortality	2629 (33.92)	55.18 (53.11 to 57.33)	2217 (28.61)	47.48 (45.54 to 49.50)	1.16 (1.09 to 1.22)
**Patients with CKD (*n* = 30 028)^b^**					
Composite CVD events	826 (19.82)	47.49 (44.36 to 50.84)	645 (15.48)	35.03 (32.43 to 37.84)	1.35 (1.22 to 1.50)
All-cause mortality	1712 (41.07)	85.65 (81.69 to 89.80)	1404 (33.69)	68.95 (65.44 to 72.65)	1.24 (1.16 to 1.33)
**Patients with HF (*n* = 12 646)^c^**					
Composite CVD events	438 (17.63)	49.86 (45.41 to 54.76)	420 (16.90)	44.37 (40.32 to 48.82)	1.12 (0.98 to 1.28)
All-cause mortality	1327 (53.40)	132.05 (125.13 to 139.35)	1222 (49.18)	113.91 (107.69 to 120.48)	1.16 (1.07 to 1.25)

a *Before propensity score matching: ADR group* n *= 7801, control group* n *= 132 683; after propensity score matching: ADR group* n *= 7750, control group* n *= 7750.*

b *Before propensity score matching: ADR group* n *= 4223, control group* n *= 26 609; after propensity score matching: ADR group* n *= 4168, control group* n *= 4168.*

c *Before propensity score matching: ADR group* n *= 2544, control group* n *= 10 102; after propensity score matching: ADR group* n *= 2485, control group* n *= 2485. ADR = adverse drug reaction. aHR = adjusted hazard ratio. CKD = chronic kidney disease. CVD = cardiovascular disease. HF = heart failure. RAAS = renin-angiotensin-aldosterone system.*

### Treatment-pattern changes following ACEI/ARB-related ADRs

Treatment-pattern changes in the year following the ADR are summarised in Table 4. Half (*n* = 4333, 50.19%) of the patients continued using ACEIs/ARBs, including switching from ACEIs to ARBs (*n* = 2228, 51.42%), continuing ACEIs (*n* = 921, 21.26%), continuing ARBs (*n* = 204, 4.71%), and switching from ACEI and ARB combinations to single ACEI/ARB drugs (*n* = 980, 22.62%). Overall, 13.17% of patients with ADRs used dual ACEI/ARB combinations, compared with only 1.22% of patients without ADRs on dual ACEI/ARB combinations. Most (*n* = 980, 86.19%) of them switched to single ACEI/ARB, with or without other antihypertensive drugs. The remaining half of the patients (*n* = 4301, 49.81%) discontinued ACEI/ARB use; the majority (*n* = 3695, 85.91%) were prescribed other antihypertensive drugs only, and a small percentage (*n* = 606, 14.09%) discontinued all antihypertensive drugs altogether. Cox regression analysis showed that the continued use of ACEIs/ARBs following ADR consultations did not lower the risk of CVD events (aHR 0.95, 95% CI = 0.85 to 1.05), but reduced the risk of all- cause mortality (aHR 0.88, 95% CI = 0.82 to 0.95) compared with the discontinuation of ACEIs/ARBs (Supplementary Table S2).

**Table 4.** Treatment-pattern changes following ADRs relating to use of ACEIs/ARBs

**Treatment changes following ACEI/ARB-related ADR**	**Total, *n* (%)**	**CVD primary prevention, *n* (%)**	**CVD secondary prevention, *n* (%)**
**Total**	8634	2889	5745

**Continued RAASI therapy**	4333 (50.19)	1679 (58.12)	2654 (46.20)
Switching from ACEI to ARB	2228 (51.42)	864 (51.46)	1364 (51.39)
Continuing on ACEI	921 (21.26)	232 (13.82)	689 (25.96)
Continuing on ARB	204 (4.71)	81 (4.82)	123 (4.63)
Switching from dual RAASI combination to single RAASI drug	980 (22.62)	502 (29.90)	478 (18.01)

**Cessation of RAASI therapy**	4301 (49.81)	1210 (41.88)	3091 (53.80)
Using other types of antihypertensive drugs only	3695 (85.91)	989 (81.74)	2706 (87.54)
Cessation of all antihypertensive drugs	606 (14.09)	221 (18.26)	385 (12.46)

*ACEI = angiotensin-converting enzyme inhibitor. ADR = adverse drug reaction. ARB = angiotensin receptor blocker. CVD = cardiovascular disease. RAASI = renin-angiotensin-aldosterone system inhibitor.*

### Sensitivity analysis

Similar findings were observed using the IPTW method, with ACEI/ARB-related ADRs being associated with an increased risk of subsequent CVD events and all-cause mortality in both primary and secondary prevention cohorts (Supplementary Table S3). In the secondary analysis, consistent results were observed when the window period was adjusted from 12 to 6 months: continued RAASI therapy was associated with reduced risk of all-cause mortality (aHR 0.88, 95% CI = 0.82 to 0.95) (Supplementary Table S4). Consistent findings were also observed among patients with complete ethnicity data (*n* = 68 591, 41.60%) (Supplementary Table S5). Patients with ADRs who continued using ACEIs/ARBs had an increased risk of CVD events compared with those who continued using ACEIs/ARBs and did not have ADRs (Supplementary Table S6).

## DISCUSSION

### Summary

Using longitudinal primary care medical records from 2004 to 2019, it was found that patients with ACEI/ARB-related ADR consultations had an increased risk of subsequent CVD events and all-cause mortality. The finding was relatively consistent across CVD history and different indications for ACEI/ARB use. In addition, it was found that the discontinuation of ACEIs/ARBs following an ADR was associated with an increased risk of all-cause mortality.

### Strengths and limitations

To the authors’ knowledge, this is the first study to examinine the impact of ACEI/ARB-related ADR consultations on patients’ outcomes in UK primary care. A thorough analysis was conducted with stratification based on CVD history and across different indications for ACEI/ARB use. In addition, treatment-pattern changes following the ADRs were examined, which might help to improve understanding of how ADRs are managed in a real-world setting and ascertain whether practice complies with treatment guidelines.

However, the study has several limitations. ADR-related consultations in primary care, which were identified using standardised designated codes, were used as a proxy for the ADRs. ACEI/ARB-related ADRs were observed in ∼1% of users of ACEIs/ARBs, which is a lower rate than that of previous studies (up to 3.9%);^13^^,^^14^ this may be due to variability in ADR assessment and/or recording.^26^^,^^27^ In addition, the severity of ADRs addressed in the consultations could not be identified, which might have affected the decision to continue or discontinue the medication. However, a previous systematic review estimated that the majority of ADRs in the primary care setting were of mild- to-moderate severity, compared with those requiring urgent medical care or hospitalisation.^38^ A relatively long interval was also found between the ACEI/ARB initiation date and the ADR dates in the present study, and, as such, it is possible that the ADRs occurred after an increase in ACEI/ARB dose. Nevertheless, the authors were unable to capture the dose relationship data in the study.

### Comparison with existing literature

Previous studies have reported that ADRs related to other cardiovascular drugs increased the risk of adverse cardiovascular outcomes.^20^^,^^26^ In addition, a study by Albani *et al*, which focused on patients with a history of acute coronary syndrome, showed that the intolerance to medications used for secondary CVD prevention, including ACEIs/ARBs, was independently associated with recurrent CVD events;^39^ this is consistent with the findings of the study presented here. Schmidt *et al* showed that elevated creatinine levels of ≥30% following ACEI/ARB use were associated with CVD events, mortality, and end- stage renal diseases.^40^ This echoes the findings of the current study, which indicate that closer monitoring for patients with potential ADRs is needed.

The findings of the present study show that half of the patients who experienced an ADR continued using ACEIs/ARBs and had a reduced risk of all-cause mortality compared with those who discontinued using ACEIs/ARBs. Several studies have examined the impact of ACEI/ARB discontinuation following a specific ADR.^41^^–^44 Leon *et al* showed that discontinuation of ACEI/ARB after hyperkalaemia was associated with an increased risk of mortality.^43^ Using target trial emulation, Xu *et al* also found that hyperkalaemia- related discontinuation was associated with an increased risk of adverse clinical outcomes, with the absolute risk difference for mortality being twice as high as that of CVD events.^44^ The decision to continue or discontinue ACEIs/ARBs following ADRs should be considered based on each patient’s circumstances. Additional treatment strategies are of importance to facilitate continued ACEI/ARB use following hyperkalaemia, and may include adequate monitoring, careful dosing, and the use of novel potassium binders, such as sodium zirconium cyclosilicate, which was found to be effective and well tolerated in patients with CKD, diabetes, and HF.^45^^,^^46^ Recently, UK guidance from the National Institute for Health and Care Excellence has recommended this agent for patients with advanced CKD and HF who cannot achieve an optimal dose of ACEIs/ARBs because of hyperkalaemia.^47^

The study presented here showed that more than half of patients who experienced an ACEI-related ADR switched to ARBs; this is in line with current clinical guidelines.^5^^,^^23^ When an ACEI-related ADR is confirmed or other causes have been ruled out, ACEI rechallenge — for example, using the same or other types of ACEI — is generally not recommended due to a high risk of recurrent reactions.^48^ Although a marginal risk of subsequent ADRs may still occur with the use of ARBs due to them having a generally similar pathway, several studies have reported that the tolerability of ARBs is excellent in patients with previous ACEI-related ADRs, with lower rate of discontinuation, cough, and angioedema.^49^^–^52 A Cochrane systematic review showed that the effectiveness of ARBs was found to be non-inferior compared with that of ACEIs.^53^

Since 2013, the use of dual ACEI and ARB combinations has not been endorsed because of an increased risk of ADRs, with no cardiovascular or mortality benefit.^54^^,^^55^ Existing evidence recommends ACEIs/ARBs with CCBs or a combination of two first- line drugs for high-risk patients, including patients with established CVD, renal disease, and those with markedly high baseline blood pressure.^8^^,^^56^^,^^57^ This combination showed superior efficacy with minimal ADRs for high- risk patients.^58^

### Implications for practice

This study showed that ACEI/ARB-related ADRs increased the risk of subsequent CVD events and all-cause mortality so, in clinical practice, the monitoring of patients affected with ADRs should be performed more closely to mitigate the risk of adverse clinical outcomes.

Clinical guidelines recommend scheduled monitoring of renal function and serum potassium among users of ACEIs/ARBs,^7^^,^^9^^,^^22^ but a previous study showed that only 10% of users of ACEIs/ARBs in the UK received guideline-recommended clinical monitoring.^59^ A study by Raebel *et al* focusing on patients with diabetes and CKD further showed that those users of ACEI/ARBs who received potassium monitoring were less likely to experience severe ADRs.^60^ Early identification of ADRs through guideline-recommended laboratory monitoring may help to mitigate the subsequent burden of the ADRs.

The monitoring should not only include laboratory monitoring but also medication adherence, as previous studies have reported that ADRs negatively affected medication adherence.^61^^,^^62^ In the subgroup analysis among patients who continued using ACEIs/ARBs, patients with ADRs had an increased risk of CVD events, compared with those without ADRs, indicating the importance of additional monitoring for affected patients as their medication adherence might be compromised even after the treatment has been switched and/or modified, resulting in suboptimal treatment outcomes. In chronic diseases such as hypertension, CKD, and HF, medication adherence is of utmost importance for disease control.^63^^–^65 Both medication safety and adherence should be monitored vigilantly by healthcare professionals for patients with ADRs, particularly when the evidence is apparent that those affected by ADRs may have an increased risk of untoward clinical outcomes. This additional monitoring may be incorporated in a medication review/structured medication review for patients with chronic disease by primary care providers. Additional treatment strategies may also be required.

## Data

All relevant data are in the article and Supplementary Data files.
